# Black Diaphragm Intraocular Lens in Patients with Aniridia

**DOI:** 10.18502/jovr.v15i4.7801

**Published:** 2020-10-25

**Authors:** Arjun Srirampur, Pasyanthi Balijepalli

**Affiliations:** Anand Eye Institute, Habsiguda, Hyderabad, India

Dear Sir,

We read with great interest the article by Al-Rashidi SH on black diaphragm intraocular lens (IOL) in patients with Aniridia.^[1]^ We would like to congratulate the author for the interesting and well-written paper, but we have some concerns regarding the article.

First, the author should have mentioned the site and size of the corneal incision made for the implantation of the IOL. As these IOLs are quite bulky, it would require at least a 9 mm incision to insert them into the anterior chamber. Such a large incision has the potential to damage the limbal stem cell population which may subsequently accelerate epithelial healing problems in patients with aniridia.

Furthermore, these patients are prone to develop intraocular pressure elevation in the long term which may need a glaucoma filtering surgery, therefore it would be prudent to spare the superior limbus for possible future glaucoma procedures.

Second, most previous reports on the long-term results of aniridia IOLs mention a high risk of postoperative cystoid macular edema (CME) and IOP elevation due to direct contact of the IOL haptics with the trabecular meshwork.^[2,3]^ It would have been nice if this paper could have provided details of the postoperative status stating whether any patient developed CME.

Third, as mentioned earlier, glaucoma is the most commonly reported complication of black diaphragm IOL implantation. Most previous reports state risks as high as 40%.^[4]^ But it is surprising to note that none of the patients in this study developed postoperative glaucoma in the follow-up period. On the contrary, all patients who were on anti-glaucoma medications preoperatively and did not require any medications in the postoperative period.

##  Financial Support and Sponsorship

Nil.

##  Conflicts of Interest

There are no conflicts of interest.
